# Germplasm, Breeding, and Genomics in Potato Improvement of Biotic and Abiotic Stresses Tolerance

**DOI:** 10.3389/fpls.2022.805671

**Published:** 2022-02-07

**Authors:** Jagesh Kumar Tiwari, Tanuja Buckseth, Rasna Zinta, Nisha Bhatia, Dalamu Dalamu, Sharmistha Naik, Anuj K. Poonia, Hemant B. Kardile, Clarissa Challam, Rajesh K. Singh, Satish K. Luthra, Vinod Kumar, Manoj Kumar

**Affiliations:** ^1^ICAR-Central Potato Research Institute, Shimla, India; ^2^School of Biotechnology, Shoolini University, Solan, India; ^3^ICAR-National Research Centre for Grapes, Pune, India; ^4^Department of Crop and Soil Science, Oregon State University, Corvallis, OR, United States; ^5^ICAR-Central Potato Research Institute, Regional Station, Shillong, India; ^6^ICAR-Central Potato Research Institute, Regional Station, Meerut, India

**Keywords:** biotic, abiotic, breeding, potato, genomics, omics approaches

## Abstract

Potato is one of the most important food crops in the world. Late blight, viruses, soil and tuber-borne diseases, insect-pests mainly aphids, whiteflies, and potato tuber moths are the major biotic stresses affecting potato production. Potato is an irrigated and highly fertilizer-responsive crop, and therefore, heat, drought, and nutrient stresses are the key abiotic stresses. The genus *Solanum* is a reservoir of genetic diversity, however, a little fraction of total diversity has been utilized in potato breeding. The conventional breeding has contributed significantly to the development of potato varieties. In recent years, a tremendous progress has been achieved in the sequencing technologies from short-reads to long-reads sequence data, genomes of *Solanum* species (i.e., pan-genomics), bioinformatics and multi-omics platforms such as genomics, transcriptomics, proteomics, metabolomics, ionomics, and phenomics. As such, genome editing has been extensively explored as a next-generation breeding tool. With the available high-throughput genotyping facilities and tetraploid allele calling softwares, genomic selection would be a reality in potato in the near future. This mini-review covers an update on germplasm, breeding, and genomics in potato improvement for biotic and abiotic stress tolerance.

## Introduction

Potato (*Solanum tuberosum* L.) is the third most important food crop of the world after rice and wheat. Potato suffers from various biotic and abiotic stresses, which may cause crop failure and yield loss depending on their severity. The key factors affecting potato cultivation are (a) biotic stresses including diseases like late blight, viruses, bacterial wilt, soil and tuber-borne diseases, insect-pests like aphids, whiteflies, thrips, mites, hoppers, potato tuber moths, and potato cyst nematodes (Singh et al., 2020); and (b) abiotic stresses like heat, drought, nutrient deficiency, salinity, and cold/frost stress (Handayani et al., 2019). Late blight is still the most serious disease of potato, however, in the current climate change scenario, viruses are becoming new threats especially for healthy seed production. Similarly, heat and drought stresses are major challenges in potato due to rising temperature, erratic rainfall, and drought conditions (Singh et al., 2020). Hence, management of these problems is very critical for developing climate resilient varieties through an accelerated breeding approach. Although conventional breeding has made significant progress, it is relatively slow and harnessed the limited potential of the *Solanum* gene pool (Hardigan et al., 2017). Now, the potato genome sequences (Potato Genome Sequencing Consortium, 2011) and resequence of wild/cultivated species are available publicly, such as *de novo* sequencing of two wild species namely *S. commersonii* (Aversano et al., 2015) and *S. chacoense* “M6” (Leisner et al., 2018); and resequencing of over 100 *Solanum* species (Hardigan et al., 2017; Kyriakidou et al., 2020; Tiwari et al., 2021). The rapid advancements in sequencing technologies, multi-omics approaches, genome editing, and genomic selection coupled with softwares/bioinformatics allow discovery of SNP markers, genes, and regulatory elements for breeding and also to enhance understanding of potato biology (Aksoy et al., 2015). This mini-review highlights the prospects of germplasm, breeding, and genomics in potato improvement for biotic and abiotic stresses tolerance.

## Biotic Stresses in Potato

### Late Blight and Viruses

Late blight, caused by the oomycete *Phytophthora infestans* (Mont.) de Bary, is the most devastating disease of potato crop worldwide. In the year 1845, this disease caused a complete loss of potato crops in the European countries mainly Ireland, and known as “Irish Famine.” More than 30 viruses are reported to infect potato crop, of which major viruses are *Potato virus X* (PVX), *Potato virus Y* (PVY), and *Potato leaf roll virus* (PLRV) in the world; and *Tomato leaf curl New Delhi virus-potato* (ToLCNDV) is a new problem in India. Potato viruses are transmitted by contact/mechanical (e.g., PVX) and insect vectors (e.g., PVY/PLRV), and cause mosaic or leaf curl and mixed symptoms (Singh et al., 2020).

### Soil and Tuber-Borne Diseases

Soil and tuber-borne diseases like dry rot (*Fusarium oxysporum*), charcoal rot (*Macrophomina phaseolina*) and bacterial soft rot (*Pectobacterium atrosepticum*) are the main problems involved in the post-harvest, storage, and transport of potato. Black scurf (*Rhizoctonia solani*) and common scab (*Streptomyces scabies*) deteriorate tuber appearance. Bacterial wilt (*Ralstonia solanacearum*) is also a serious disease, while wart caused by *Synchytrium endobioticum* is a problem of hilly regions like Darjeeling hills in India. These diseases are managed by using healthy seeds, disinfection by boric acid treatment, cultural practices, and crop rotation (Singh et al., 2020).

### Insect-Pests

Insect-pests such as aphids, whiteflies, thrips, white grubs, cutworms, leaf hopper, potato tuber moths, and mites infest potato crop. Aphids (*Myzus persicae*) transmit viruses in two ways i.e., persistent and circulative (PLRV), and non-persistent (PVY). Whiteflies (*Bemisia tabaci*) transmit ToLCNDV-potato virus. Thrips (*Thrips palmi*) transmit *Groundnut bud necrosis virus* and cause stem necrosis disease. Importantly, potato cyst nematodes (PCN) (*Globodera rostochiensis* and *G. pallida*) are key problems in temperate regions. Besides, other insect-pests are potato leaf hopper (*Amrasca biguttula biguttula*), white grub (*Brahmina coriacea*), cutworm (*Agrotis segetum*), potato tuber moth (*Phthorimaea operculella*), and mites (*Polyphagotarsonemus latus*) (Singh et al., 2020).

## Abiotic Stress in Potato

### Heat and Drought Stress

Heat stress is a great problem for potato crop, particularly in early planted crop and after the harvest of the main *rabi* crop under sub-tropical Indian conditions. A minimum night temperature below 20°C (day 25°C) is essential for tuber growth and development (Singh et al., 2015). Potato is mostly an irrigated crop, except in rain fed hilly regions. Therefore, all growth stages are sensitive to water availability such as germination, foliage, and root/stolon/tuber growth. Thus drought i.e., moisture deficit plays a very crucial role in determining potato yield (Dahal et al., 2019).

### Nutrient Deficiency, Salinity, and Frost/Cold stress

Nutrients are very essential for plant growth, yield, and quality of potato. Potato is a heavily fertilized crop especially for nitrogen (N), and therefore reduction of N fertilizers is necessary to save the environment and reduce the production cost (Zhang et al., 2020). Nutrient deficiency drastically affects crop growth and reduces yield. Besides, salinity is another problem due to soil or irrigation water, which causes nutrient imbalance and restricts plant growth. Frost/cold is also another issue of temperate climates, where temperatures below −2°C can result in a partial or complete loss of crop (Ahmed et al., 2020).

## Germplasm, Mapping, and Breeding

### Potato Genetic Resources

Potato belongs to the genus *Solanum* (family: Solanaceae), which contains over 2,000 species, of which nearly 235 are tuber bearing potato species, where 73% are diploids (2*x*), 4% triploids (3*x*), 15% tetraploids (4*x*), and 8% pentaploids (5*x*)/hexaploids (6*x*) (Hawkes, 1990). The cultivated potato (*S. tuberosum* ssp. *tuberosum*) is a tetraploid (2*n* = 4*x* = 48). Potatoes are classified into four major groups (i) *S. tuberosum* group Andigenum of upland Andean genotypes (2*x*/3*x*/4*x*), and *S. tuberosum* group Chilotanum of lowland Chilean landraces (4*x*), (ii) *S. ajanhuiri* (2*x*), (iii) *S. juzepczukii* (3*x*), and (iv) *S. curtilobum* (5*x*) (Spooner et al., 2014). These species belong to different endosperm balance numbers (EBNs) like 1EBN (2*x*), 2EBN (2*x*/4*x*), and 4EBN (4*x*/6*x*), where hybridization within the same EBN species is successful but not with different EBN species (Hawkes, 1990). Over 98,000 potato accessions are conserved *ex situ* (*in vitro*), of which 80% are maintained in 30 key collections worldwide (FAO, 2010). To harness the potential of diverse species, a wide range of genetic variation has been recorded and deployed through breeding and ploidy manipulation techniques for potato improvement.

### Linkage and Association Mapping

Gene mapping is important for molecular breeding. The complex tetrasomic inheritance, acute inbreeding depression, and high heterozygosity of potato complicate its genetic mapping. Linkage mapping is the genetic association of traits with segregating alleles of molecular markers in a defined mapping population. The first linkage map was reported in 1988 using tomato RFLP (restriction fragment length polymorphism) markers in diploid species (*S. tuberosum* group Phureja/Tuberosum) (Bonierbale et al., 1988). Then uncounted PCR-based molecular markers like simple sequence repeat (SSR), amplified fragment length polymorphism (AFLP), and diversity array technology (DArT) were applied for mapping. Over 10,000 AFLP markers were used to create an ultra-high-density (UHD) genetic and physical map of potato (Van Os et al., 2006), which was used in the potato genome sequencing. Sharma et al. (2013) constructed a dense genetic and physical map for a diploid backcross progeny using 2,469 markers (SSR/AFLP/DArT/SNP). Numerous genes/QTLs have been mapped in potato for various traits like late blight resistance (Hein et al., 2009) and drought stress (Anithakumari et al., 2012). On the contrary to linkage mapping, association mapping identifies genes/QTLs associated with phenotypic variation in a natural population based on the historical recombination and linkage disequilibrium (Flint-Garcia et al., 2003). In potato, diploid/tetraploid clones have been utilized in association mapping for several agronomic traits (D’hoop et al., 2014), particularly resistance to late blight (Gebhardt et al., 2004) and *Verticillium* wilt (Simko et al., 2004) to name a few.

### Marker-Assisted Selection

Over 40 traits are considered to be important in potato breeding. Conventional breeding is a time consuming process mainly due to several years of field evaluation and clonal selection. Hence, identification of tightly linked markers with a target gene for a trait is considered to be ideal for MAS. MAS allows a significant decrease in field exposures by selection in the early stage, and thereby reduces field exposures and breeding cycles. In potato, a considerable number of linked markers have been developed and deployed mainly for simply inherited traits like late blight, viruses, and potato cyst nematode resistance (Ramakrishnan et al., 2015). However, meager information is available on MAS for complex traits like yield, nutrient use efficiency, heat, drought, and cold stress.

## Progress in Genomics-Led Potato Improvement

### Potato Genome Sequencing/Resequencing

**FIGURE 1:**
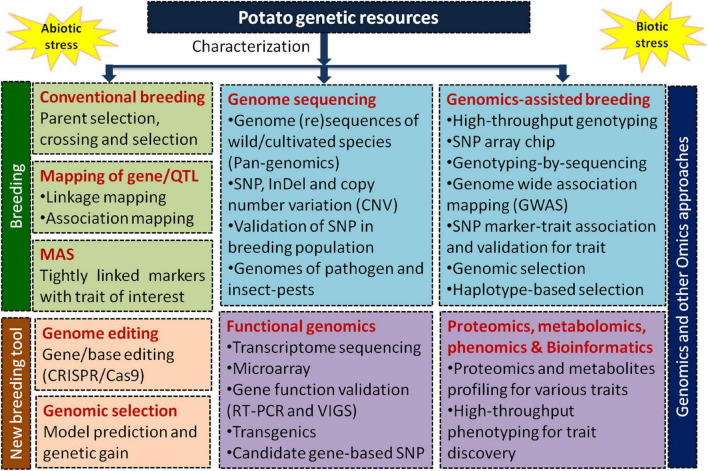
A schematic presentation of different approaches used for genetic enhancement and improvement of potato under various biotic and abiotic stresses applying breeding and modern genomics approaches like genome sequencing, functional genomics, genomics-assisted breeding through high-throughput genotyping by SNP markers, omics (transcriptomics, proteomics, metabolomics, and ionomics), high-throughput phenotyping, genome editing, and genomic selection.

In 2011, the Potato Genome Sequencing Consortium (PGSC),—formed by 26 international institutes belonging to 14 countries—successfully deciphered the potato genome (840 Mb) containing 39,031 protein-coding genes using a homozygous doubled monoploid (DM 1-3 516 R44) of *S. tuberosum* group Phureja (2*n* = 2*x* = 24) (Potato Genome Sequencing Consortium, 2011)^1^. Later Sharma et al. (2013) improved the DM potato assembly with a more accurate arrangement of scaffolds and pseudomolecules. Recently, a chromosome-scale long-read reference assembly has been constructed (Pham et al., 2020). By now over 100 potato species have been sequenced/re-sequenced mostly using Illumina platforms like wild *S. commersonii* (Aversano et al., 2015), tuber-bearing *Solanum* species (Hardigan et al., 2017), *S. chacoense* “M6” (Leisner et al., 2018), *S. pinnatisectum*-derived somatic hybrid (Tiwari et al., 2021), and cultivated potato taxa using Illumina and long-read (PacBio) technologies (Kyriakidou et al., 2020; Table 1). The rapid advancement in sequencing and bioinformatics has spurred innovation in discovery of new genes/markers/haplotypes to enable better understanding of potato biology (Zhou et al., 2020). Figure 1 illustrates different approaches used in potato germplasm, breeding, and genomics-led improvement for biotic and abiotic stresses tolerance.

**TABLE 1:** A few recent examples of application sequencing and multi-omics technologies in potato for biotic and abiotic stress resistance/tolerance.

Application	System	Traits/objectives	References
Genome sequencing	Illumina HiSeq (and PacBio in some species)	Genome sequencing and structural variation in many *Solanum* species	Hardigan et al., 2017; Kyriakidou et al., 2020
		*S. checoense* “M6” genome	Leisner et al., 2018
		*S. commersonii* genome	Aversano et al., 2015
Genome-wide genetic diversity and GWAS	22K SNP array	Construction of core collection	Pandey et al., 2021
	20 K SNP array	Population structure, LD and SNP/haplotypes	Vos et al., 2017
	12K SNP array	Population structure of CIP accessions	Ellis et al., 2018
	8.3 K SNP array	Population structure and LD	Berdugo-Cely et al., 2017
	RenSeq/GenSeq	Late blight and nematode resistance	Strachan et al., 2019
	20K SNP array	Wart disease resistance	Prodhomme et al., 2020
	12 K SNP array	Common scab resistance	Yuan et al., 2020
	8.3K SNP array	Late blight resistance	Mosquera et al., 2016
Genomic selection	8.3k SNP array	Late blight resistance	Stich and Inghelandt, 2018
	8.3k SNP array	Late blight and common scab resistance	Enciso-Rodriguez et al., 2018
Transcriptomics	Illumina	Late blight, bacterial wilt,	Cao et al., 2020
	HiSeqTM2500	and PVY resistance	
	Illumina HiSeq2500	Common scab resistance	Fofana et al., 2020
	Ion torrent	Colorado potato beetle resistance	Bastarache et al., 2020
	Illumina	Potato cyst nematode	Kochetov et al., 2020
	NextSeq500	resistance	
	Illumina HiSeq × Ten	Salt stress	Li et al., 2020
	Illumina NextSeq	Drought stress	Moon et al., 2018
	Illumina HiSeq 4000	Drought stress	Chen et al., 2019
	Illumina HiSeq-2000	Heat stress	Tang et al., 2020
	Illumina	Nitrogen stress	Tiwari et al., 2020b; Zhang et al., 2020
	NextSeq500	Nitrogen stress	
	Illumina HiSeq 4000		
Proteomics	iTRAQ	Late blight resistance	Xiao et al., 2020
	iTRAQ	Bacterial wilt resistance	Wang et al., 2021
Metabolomics	LC-MS/MS	Potato virus A resistance	Rajamaki et al., 2020
	GC-MS	Salt stress	Hamooh et al., 2021
	LC-ESI-Q-TOF-MS/MS	Nitrogen stress	Jozefowicz et al., 2017
Transcriptomics and metabolomics	Illumina HiSeq 4000, LC-MS	Heat stress	Liu et al., 2021
Proteomics and metabolomics	2-DE	Cold stress	Li et al., 2021
	LC-ESI-MS/MS		
Phenomics (HTP)	X-ray computed tomography (CT)	Heat and drought stress	Harsselaar et al., 2021
	RGB camera and LED light system	Drought stress	Musse et al., 2021
	Unmanned aerial vehicle	Plant height and canopy cover	Colwell et al., 2021
Genome editing	CRISPR/Cas13a	PVY resistance	Makhotenko et al., 2019; Zhan et al., 2019
	CRISPR/Cas9		

*LD, linkage disequilibrium; CIP, International Potato Center; GWAS, Genome-Wide Association Studies; htp, high-throughput phenotyping.*

### Multi-Omics Approaches

Functional genomics allows the mining of genes for trait of interest through transcriptome analysis like RNA sequencing and microarray. Besides structural genomics, other omics approaches are transcriptomics (genes), proteomics (proteins), metabolomics (metabolites), phenomics (high-throughput phenotyping), and ionomics (mineral ions). The aims of multi-omics approaches are to acquire comprehensive and integrated understanding of biological processes (system biology) to identify various biological players/genes/regulatory elements underlying the traits like heat and drought stress (Aksoy et al., 2015). Numerous studies have been performed on transcriptomics in potato such as heat (Tang et al., 2020), drought (Moon et al., 2018; Chen et al., 2019), salinity (Li et al., 2020), and nitrogen deficiency (Tiwari et al., 2020a,b) but limited work has been carried out on proteomics, metabolomics, and ionomics (Hong et al., 2016; Boguszewska-Mańkowska et al., 2020). A few recent research works on multi-omics on biotic/abiotic stresses are mentioned in Table 1.

### Genome-Wide Genetic Diversity and Association Studies Using High-Throughput Genotyping

High-Throughput Genotyping (HTG) is an essential requirement for genome-wide research on genetic diversity and association studies. First, genotyping-by-Sequencing (GBS) is a now popular method of HTG in crops including potato (Uitdewilligen et al., 2013; Bastien et al., 2018). GBS has been applied effectively in genome-wide studies in potato on genetic diversity and population structure (Pandey et al., 2021), QTL mapping (Schönhals et al., 2017), and SNP discovery (Caruana et al., 2019). Secondly, the SNP array-based HTG system has already been developed and applied in potato for population structure and SNP discovery using 20K SNP array (Vos et al., 2015, 2017), 22K SNP array for starch phosphorylation (Khlestkin et al., 2019), and 12K SNP array (Illumina) for genetic diversity in the genbank of the International Potato Centre, Peru (Ellis et al., 2018). Moreover, 8.3K SNP potato array has been demonstrated in studies on *Synchytrium endobioticum* resistance (Obidiegwu et al., 2015), genetic diversity (Berdugo-Cely et al., 2017), and physical mapping of yield and quality traits (Schönhals et al., 2017).

Genome-Wide Association Studies (GWAS) or linkage disequilibrium (LD) mapping is a family-based mapping approach to identify linked markers with the trait of interest in a diverse population structure. GWAS is more useful in a diverse germplasm which offers new perspectives toward the discovery of new genes and alleles especially for complex traits. The software STRUCTURE is very popular among scientific communities, and GWASpoly has been developed for tetraploid potato (Rosyara et al., 2016). GWAS has been applied in potato for QTLs/genes via LD mapping using 20K SNP array (Vos et al., 2017), wart resistance using 20K SNP array (Prodhomme et al., 2020) and common scab resistance using 12K SNP array (Yuan et al., 2020). Likewise, 8.3K SNP array has been used in LD mapping for phenotype, yield, and quality traits (Sharma et al., 2018), late blight resistance (Mosquera et al., 2016), and genetic diversity in 809 andigenum Colombian accessions (Berdugo-Cely et al., 2017). Applications of SNP array in potato for biotic and abiotic stress traits are summarized in Table 1.

### Genomic Selection

Genomic selection (GS) or genome-wide selection or genomics-assisted breeding is a strategy to predict breeding model at whole-genome level for both simple and complex inherited traits. Therefore, partitioning of genetic variance and genome wide prediction with allele doses is very important in tetraploid potato (Endelman et al., 2018). GS allows the integration of phenotyping and HTG data of a training population (both genotyped and phenotyped) with a targeted breeding population (genotyped only) for the prediction of genomic models to select superior clones based on the genomic estimated breeding value (GEBV). GS accelerates the breeding cycle with an increase in genetic gain per unit time. Unlike animals and cereals, the application of GS is very limited in tetraploid potato (Caruana et al., 2019) and has been demonstrated recently for late blight and common scab resistance (Enciso-Rodriguez et al., 2018; Stich and Inghelandt, 2018). The advancement in sequencing, softwares, HTG, HTP, and marker-trait association can reduce the breeding cycle from over 10 to as few as 4 years to increase the genetic gain in potato (Slater et al., 2014; Table 1).

### High-Throughput Phenotyping

Conventional phenotyping is often slow, has limited phenotyping capability, and mostly relies upon destructive sampling. Hence, modern High-Throughput Phenotyping (HTP) or phenomics is an automated precision phenotyping system allowing identification of key traits associated with phenotypic variation under different growth conditions. HTP is usually based on automation, sensors, high resolution imaging cameras (RGB, multi/hyperspectral and thermal sensors), unmanned aerial vehicle (UAV) and robotics to record real-time images and hardwares/softwares to analyze data from field or controlled growth chamber^2^. HTP enables measurement of phenotype, yield and its contributing traits, and physiological processes under stress such as photosynthesis, nutrient uptake and transport with precision and accuracy in a large set of genotypes with non-destructive sampling, for example the LemnaTec Scanalyzer 3D platform (LemnaTech GmbH, Germany). HTP has been applied in potato for phenology study in field (Prashar and Jones, 2014), heat and drought (Harsselaar et al., 2021), drought (Musse et al., 2021), and canopy cover using UAV (Colwell et al., 2021). Examples of HTP in heat and drought stress in potato are mentioned in Table 1.

### Genome Editing

Genome editing is a powerful technology to create new variation in the genome with desirable gene combinations. Earlier sequence-specific nucleases (SSNs) methods like Zinc Finger Nucleases (ZFNs) and Transcription Activator-Like Effector Nucleases (TALENs) were used. Now, Clustered Regularly Interspaced Short Palindromic Repeats (CRISPR)/CRISPR-associated protein 9 (Cas9) is the most widely used genome editing tool, which is an RNA-guided approach to target DNA/RNA sequences. CRISPR/Cas9 has revolutionized the plant research for multiple traits due to its ease in use, multiplexing capability, cost-effectiveness, and high efficiency. Although, in potato highly heterozygous and tetrasomic inheritance have complicated its deployment (Butler et al., 2015; Andersson et al., 2017) but found effective for PVY resistance using CRISPR/Cas9 (Makhotenko et al., 2019) and CRISPR/Cas13a (Zhan et al., 2019). Additionally, CRISPR/Cas9 has been demonstrated in potato for various other traits like cold-induced sweetening, glycoalkaloid content, homozygous mutants generation, *acetochalactate synthase 1* and *granule bound starch synthase* genes (Nadakuduti et al., 2018; Dangol et al., 2019; Table 1).

## Concluding Remarks

Biotic and abiotic stresses are major limiting factors of yield reduction in potato. Management of these stresses are more challenging under the climate change scenario due to emergence of new strains of pathogens and insect-pests, and erratic nature of environmental factors. Potato improvement through genomics-aided methods is essential to shorten the breeding cycle to develop new varieties. Earlier, conventional breeding, bi-parental linkage mapping, and MAS have been successfully demonstrated in potato. The potato genome sequencing and resequencing of *Solanum* species allow discovery of genes, markers and other regulatory elements to provide better understanding of the crop. Now, with the unprecedented advancement in sequencing technologies, genomes of *Solanum* species (pan-genomics), multi-omics for system biology approach (transcriptomics, proteomics, metabolomics, and ionomics), HTG by GBS and SNP array, HTP for precision phenotyping, GWAS and genomic selection would play crucial roles in genomics-led improvement of potato in the near future. There is an immense potential of genome editing for rapid breeding of climate resilient varieties resistant/tolerant to biotic and abiotic stresses. Nonetheless, the availability of an efficient CRISPR/Cas system, target gene selection, plant transformation, and off target mutants would be some challenges in tetraploid crop. Overall, designs of potato that apply genomics, particularly genome editing and genomic selection, and other omics are inevitable in the future.

## Author Contributions

JT conceived idea and wrote manuscript. JT, TB, RZ, NB, DD, SN, HK, CC, SL, and VK performed research and literature collection. RS, AP, and MK edited the manuscript. All authors approved the manuscript.

## Conflict of Interest

The authors declare that the research was conducted in the absence of any commercial or financial relationships that could be construed as a potential conflict of interest.

## Publisher’s Note

All claims expressed in this article are solely those of the authors and do not necessarily represent those of their affiliated organizations, or those of the publisher, the editors and the reviewers. Any product that may be evaluated in this article, or claim that may be made by its manufacturer, is not guaranteed or endorsed by the publisher.

## Footnotes

1
http://solanaceae.plantbiology.msu.edu/pgsc_download.shtml

2
http://www.plantaccelerator.org.au/
